# Prognostic role of serum cytokeratin 19 fragments in advanced non-small-cell lung cancer: association of marker changes after two chemotherapy cycles with different measures of clinical response and survival

**DOI:** 10.1038/sj.bjc.6604157

**Published:** 2007-12-18

**Authors:** B Nisman, H Biran, N Heching, V Barak, N Ramu, I Nemirovsky, T Peretz

**Affiliations:** 1 Department of Oncology, Hadassah University Hospital, Jerusalem, Israel; 2 Lung Cancer Unit, Division of Oncology, Sheba Medical Center, Tel Hashomer, Israel

**Keywords:** CYFRA 21-1, non-small-cell lung carcinoma, clinical response, survival prediction

## Abstract

Prognostic implication of serum cytokeratin 19 fragments (CYFRA 21-1) was explored in 60 advanced NSCLC patients, whereas in 45 patients assessable for serological response a ⩾35% CYFRA 21-1 decline after two chemotherapy cycles was strongly associated with non-progression (NP), defined as a sum of objective response (OR)+stable disease (*P*<0.0001) and survival (*P*=0.0002). Association of OR with survival was not significant. In multivariate survival analysis, ⩾35% marker decline and radiological NP status were found as major determinants of prolonged survival with RR: 0.37 (*P*=0.01) and 0.63 (*P*=0.01), respectively. In advanced NSCLC patients, NP reflects therapeutic efficacy better than traditional OR. CYFRA 21-1 ⩾35% decline seems to be a reliable surrogate marker of treatment efficacy in terms of survival.

Combination chemotherapy for advanced non-small-cell lung cancer (NSCLC) is clinically beneficial in only about half of the treated naive patients, and most responses are partial.

The decision whether to continue or to stop therapy is traditionally guided by imaging-based tumour response evaluation, which is regarded a surrogate marker of clinical benefit. Assessment by structural imaging has known limitations and also may have a poor correlation with pathologic response in NSCLC (Martini *et al*, 1988).

Furthermore, it has been shown that objective response (OR) is not a wholly reliable prognosticator of survival (Gandara *et al*, 1999). In this context, the diagnostic and prognostic potential of biochemical markers, such as circulating cytokeratin 19 fragments (CYFRA 21-1), in monitoring antineoplastic therapy has been investigated (Ebert and Muley 1999, Ardizzoni *et al*, 2006).

In the present study, we compared CYFRA 21-1 change with clinical response after two chemotherapy cycles in order to find out which one correlates better with patients' survival.

## PATIENTS AND METHODS

### Patients

This prospective, IRB approved, study included 60 chemotherapy-naive patients enrolled between 1 January 1998 and 1 July 2003. Patient characteristics are presented in Table 1. The median age was 61 years (37–77 years). All patients received a minimum of two, and up to eight (median 5) chemotherapy cycles (=courses). Specific regimens are presented in Table 1. CT scans were performed every two cycles. All patients had measurable disease. A functional state was graded by the Eastern Cooperative Oncology Group (ECOG) performance status (PS) scale.

Two response categories were explored: OR (complete response (CR)+partial response (PR)) and non-progression – NP (CR+PR+stable disease (SD)). Patients achieving either OR or NP were classified as responders, whereas those with no response (NR) (SD+progressive disease (PD)) or PD, respectively, were considered non-responders.

### Methods

Serum samples were obtained before the start and after the second chemotherapy course at the time of clinical response evaluation and stored at −80°C until analysis. Cytokeratin 19 fragments were measured by immunoradiometric ELSA-CYFRA 21-1 kit (CIS Bio International, Gif-sur Yvette, France). The cut-off value was 3.2 ng ml^−1^. It provided 95% specificity in the group of patients with benign lung diseases (Nisman *et al*, 1998). From two serum samples, pretreatment and that obtained after two cycles of chemotherapy, at least one was required to be above the normal cut-off (3.2 ng ml^−1^) to have the CYFRA 21-1 change assessable disease.

### Statistical analysis

Non-parametric tests were used for comparisons between numeric variables, the Wilcoxon signed-rank test for CYFRA 21-1 before and after treatment and Mann–Whitney for independent groups. Spearman's correlations were reported. Associations between categorical variables were evaluated with the *χ*^2^-test. The Youden index was used to identify the optimal decline in CYFRA 21-1 for the diagnosis of NP or OR (Armitage, 1971). Univariate analyses of the prognostic impact of various factors on survival from response evaluation (i.e., landmark time of 6 weeks) to death or last follow-up used the Kaplan–Meier method and log-rank test. Variables statistically significant (*P*<0.05) in the univariate steps were entered into multivariate Cox regression models (Cox, 1972). The proportional hazard assumption was examined in log minus log plots and found acceptable. A value of *P*<0.05 was considered significant.

## RESULTS

### Whole study population (*N*=60)

Pre- and post-treatment, two-course median CYFRA 21-1 were 5.5 ng ml^−1^ (range 0.89–88.6 ng ml^−1^) and 2.2 ng ml^−1^ (range 0.28–64.1 ng ml^−1^), respectively. Between two consecutive measurements, CYFRA 21-1 showed a significant decline (*P*=0.001).

Median marker change in OR (−76%) was close to that of SD (−58%) (P=0.7), but significantly higher than that in PD (5%) (*P*=0.003).

In OR and NR subgroups, the median CYFRA 21-1 changes after two courses did not differ significantly (−76 and −34%, respectively, *P*=0.1); whereas in NP, the median change was significantly larger than in PD (−68 and 5%, respectively, *P*=0.001).

CYFRA 21-1 levels were above the cut-off limit (3.2 ng ml^−1^) in 43 (71.7%) and in 24 (40%) of 60 patients pre- and post-treatment two courses, respectively. In only 2 out of 60 patients (3.3%), elevation greater than cut-off level occurred during initial therapy.

Overall median survival from the landmark time was 12.7 months. Median survival of either OR and or SD patients was significantly longer than that of PD patients (16.3 and 14 *vs* 6.1 months; *P*<0.0001 and *P*=0.0005, respectively), whereas OR and SD patients were similar (*P*=0.69). Non-progression patient's median survival was 15.1 months.

Table 2 depicts patient characteristics, biochemical parameters and the response category, associated with survival. Association of OR with survival did not reach statistical significance (*P*=0.06). When pretreatment significant parameters were included in the Cox model, an independent prognostic status was retained for PS (relative risk (RR): 2.0; 95% CI 1.1–3.5; *P*=0.03), stage (RR: 1.9; 95% CI 1.1–3.4; *P*=0.02) and pre-treatment CYFRA 21-1 (1-CYFRA 21-1) (RR: 0.5; 95% CI 0.3–0.9; *P*=0.02). When, however, the aforementioned three variables were complemented by post-treatment two courses CYFRA 21-1 (2-CYFRA 21-1) and NP, only the latter two stayed independent with a RR: 0.40; 95% CI 0.19–0.84; *P*=0.01 and RR: 0.55; 95% CI 0.39–0.76, *P*<0.001, respectively.

### Subgroup of patients with CYFRA 21-1 change assessable disease (*N*=45)

According to the used criterion presented in the method section, 45 patients were found to have disease assessable for marker response. In this subgroup, the CYFRA 21-1 declines, best correlating with NP and OR corresponded to a ⩾35 and ⩾62% reduction in CYFRA 21-1 after two chemotherapy courses (marker response-35 and marker response-62, respectively).

The cut-off ⩾35% was 84.6% sensitive and 79% specific in the diagnosis of NP (accuracy, 82.2%). Marker response-35 was achieved in 22 out of 26 NP patients but only in 4 out of 19 PD patients (*P*<0.0001). Response-35 conferred a longer survival (14.0 months *vs* 6.1 months, *P*=0.0002).

Marker response-62 was 90% sensitive and 60% specific with regard to OR (accuracy, 66.7%). It occurred in 9 out of 10 OR and in 14 out of 35 NR patients (*P*=0.02). Patients who had ⩾62% CYFRA 21-1 reduction survived 14 *vs* 6.6 months for those who had a lesser decrease (*P*=0.02).

Non-progression patients showed a far better survival as compared to PD patients (14.0 *vs* 6.1 months, *P*<0.0001). Survival difference between OR and NR patients was not significant (*P*=0.3).

When PS, marker response-35 and NP were included in the Cox model, only marker response-35 (RR: 0.4; 95% CI 0.2–0.8; *P*=0.01) and NP (RR: 0.6; 95% CI 0.4–0.9; *P*=0.01), but not PS (RR: 1.8; 95% CI 0.9–3.5; *P*=0.11), remained significant determinants of survival.

We investigated the prognostic role of CEA changes during chemotherapy as well. In the univariate analysis, CEA response (⩾35% decline) was found to be associated with prolonged survival (14.0 *vs* 8.8 months; *P*=0.04). Being included in the Cox model with PS and NP, CEA response, however, did not retain significance (*P*=0.78).

## DISCUSSION

This is the first study comparing the prognostic impact of two clinical response category groups, namely OR and NP, with two corresponding CYFRA 21-1 responses differing in magnitude of decline.

Our study together with a previous report (Lara *et al*, 2006) questions the validity of OR as a survival indicator. It shows the survival of SD standing more close to OR than to PD patient categories, suggesting that a stable disease by NSCLC chemotherapy contributes significantly to patients' survival. Thus, NP (PR+SD) appears to measure treatment efficacy more accurately than traditional OR.

In the study, we found that chemotherapy-induced CYFRA 21-1 changes had a strong association with both radiological response (OR, SD and PD) and survival. Furthermore, the CYFRA 21-1 change at SD were closer to that in OR than in PD, corroborating Ebert and Muley (1999) study of inoperable NSCLC patients.

In addition to known unfavourable prognostic implication of high pretreatment CYFRA 21-1 in advanced NSCLC (Pujol *et al*, 2004), we have observed that therapy-induced 2-CYFRA 21-1 impacts survival even more. Inclusion of 2-CYFRA 21-1 and NP in the Cox model replaced pretreatment CYFRA 21-1, PS and stage of disease, becoming the major determinants of survival with relative risks 0.40 and 0.55, respectively.

In the subgroup of 45 patients with serologically assessable disease, the marker declines best associated with clinical OR and NP outcomes comprised 62 and 35%, respectively.

Clinically, NP is more meaningful than OR; interestingly, marker response-35 reflected NP and survival better than marker response-62 for OR and survival. We suggest that NP, incorporating SD, really groups the patients benefiting from therapy. These two parameters have an independent impact as prognostic factors for survival.

High accuracy of the marker response-35 in diagnosis of NP and its strong association with survival makes it possible to use it as a reliable surrogate marker of treatment efficacy in advanced NSCLC, and a tool to be considered in the design of future NSCLC trials.

We are aware of the small cohort of patients in this study, which limits the power of multivariate analyses and thus warrant validation and refinement by larger scale prospective trials.

## Figures and Tables

**Table 1:** Patient and disease characteristics

**Characteristics**	***n***
*Gender*	
Males/Females	43/17
	
*Age* (*years*)	
⩽61/>61	32/28
	
*Stage*	
IIIA/IIIB/IV	5/18/37
*ECOG PS*	
0–1/2	41/19
	
*Tumour histology*	
Adenocarcinoma	29
Squamous	9
Large cell	17
Unclassified NSCLC	5
	
*First line chemotherapy*	
Cisplatin+gemcitabine	29
Carboplatin+paclitaxel	23
Carboplatin+etoposide	6
Others	2
	
*Clinical response*	
CR/PR/SD/PD	1/16/22/21
	
*Status at last follow-up*	
Alive/dead of tumour	1/59

ECOG PS=Eastern Cooperative Oncology Group performance status; NSCLC=non-small-cell lung cancer; CR=complete response; PR=partial response; SD=stable disease; PD=progressive disease.

**Table 2:** Univariate survival analyses in 60 patients with NSCLC (only variables with *P*<0.1 were included)

**Characteristics**	***N***	**MS (months)**	**95% CI**	***P*-value log rank**
*Stage*				
IIIA-IIIB	23	13.7	11.7–15.7	0.04
IV	37	8.9	3.9–13.9	
				
*ECOG PS*				
⩽1	41	13.0	10.9–15.1	0.02
>1	19	7.5	1.4–13.6	
				
*NP*				
Yes	39	15.1	13.0–17.2	<0.0001
No	21	6.1	2.7–9.5	
				
*OR*				
Yes	17	16.3	11.0–21.6	0.06
No	43	8.8	3.9–13.6	
				
*1-CYFRA 21-1* (*ng* *ml*^−*1*^)				
⩽3.2	17	19.5	11.4–27.6	0.01
>3.2	43	10.6	5.8–15.4	
				
*2-CYFRA 21-1* (*ng* *ml*^−*1*^)				
⩽3.2	36	15.1	13.5–16.7	<0.0001
>3.2	24	6.2	5.2–7.2	

1-CYFRA 21-1=pretreatment CYFRA 21-1; 2-CYFRA 21-1=CYFRA 21-1 after two cycles of chemotherapy; CI=confidence interval; ECOG PS=Eastern Cooperative Oncology Group performance status; MS=median survival time; NSCLC=non-small-cell lung cancer; NP=non-progression; OR=objective response.
